# Niacin-mediated Gene Expression and Role of NiaR as a Transcriptional Repressor of *niaX, nadC*, and *pnuC* in *Streptococcus pneumoniae*

**DOI:** 10.3389/fcimb.2017.00070

**Published:** 2017-03-09

**Authors:** Muhammad Afzal, Oscar P. Kuipers, Sulman Shafeeq

**Affiliations:** ^1^Department of Molecular Genetics, Groningen Biomolecular Sciences and Biotechnology Institute, University of GroningenGroningen, Netherlands; ^2^Department of Bioinformatics and Biotechnology, Government College UniversityFaisalabad, Pakistan; ^3^Department of Microbiology, Tumor and Cell Biology, Karolinska InstitutetStockholm, Sweden

**Keywords:** niacin, NiaR, Pneumococcus, *niaX*, *nadC*, *pnuC*

## Abstract

NAD (Nicotinamide Adenine Dinucleotide) biosynthesis is vital for bacterial physiology and plays an important role in cellular metabolism. A naturally occurring vitamin B complex, niacin (nicotinic acid), is a precursor of coenzymes NAD and NADP. Here, we study the impact of niacin on global gene expression of *Streptococcus pneumoniae* D39 and elucidate the role of NiaR as a transcriptional regulator of *niaX, nadC*, and *pnuC*. Transcriptome comparison of the D39 wild-type grown in chemically defined medium (CDM) with 0 to 10 mM niacin revealed elevated expression of various genes, including *niaX, nadC, pnuC, fba, rex, gapN, pncB, gap, adhE*, and *adhB2* that are putatively involved in the transport and utilization of niacin. Niacin-dependent expression of these genes is confirmed by promoter *lacZ-*fusion studies. Moreover, the role of transcriptional regulator NiaR in the regulation of these genes is explored by DNA microarray analysis. Our transcriptomic comparison of D39 Δ*niaR* to D39 wild-type revealed that the transcriptional regulator NiaR acts as a transcriptional repressor of *niaX, pnuC*, and *nadC*. NiaR-dependent regulation of *niaX, nadC*, and *pnuC* is further confirmed by promoter *lacZ-*fusion studies. The putative operator site of NiaR (5′-TACWRGTGTMTWKACASYTRWAW-3′) in the promoter regions of *niaX, nadC*, and *pnuC* is predicted and further confirmed by promoter mutational experiments.

## Introduction

Bacteria can trigger transcriptional and phenotypic programs to synchronize an adaptive response in reaction to environmental fluctuations or stresses (Edwards et al., 2013). This not only relies on the number of virulence factors it possesses, but also on the proper use of nutrients available in the human niches (Phillips et al., 1990; Titgemeyer and Hillen, 2002). A number of important vitamins and co-factors are required by bacteria to survive and grow successfully. *Streptococcus pneumoniae*, a major Gram-positive human pathogen and nasopharyngeal colonizer, encounters different environmental factors and has to fine-tune its gene expression accordingly (Bogaert et al., 2004; Kadioglu et al., 2008).

Niacin (nicotinic acid), a naturally occurring vitamin B complex, is a precursor of coenzymes NAD and NADP, and plays an important role in electron transfer during metabolic processes (Wei et al., 2014). Niacin has long been used for the treatment of lipid disorders and cardiovascular disease (Wei et al., 2014). It can regulate the activity of microbial two-component systems and, subsequently, modulate the genes and phenotypes that are controlled by these regulatory proteins (McPheat et al., 1983). Particularly, niacin has been reported to repress the expression of many genes including virulence factors in *Bordetella pertussis*, such as pertussis toxin, adenylate cyclase toxin, and filamentous hemagglutinin (Schneider and Parker, 1982; McPheat et al., 1983; Cotter and DiRita, 2000; Cummings et al., 2006). Furthermore, the two-component system BvgA/BvgS, which is known to have a role in the regulation of virulence and colonization, becomes inactive in *B. pertussis* when niacin is present in the medium (Miller et al., 1989). Similarly, the *Escherichia coli* EvgA/EvgS system that confers multidrug resistance and acid tolerance is regulated by niacin (Masuda and Church, 2002, 2003; Eguchi et al., 2003; Nishino et al., 2003). Both the BvgA/BvgS system of *B. pertussis* and the EvgA/EvgS system of *E. coli* are part of a family of proteins that utilize a multistep phosphor-relay to trigger their responsive pathways.

It has been proposed that in *S. pneumoniae* niacin enters the cell through NiaX and is converted to nicotinate (nicotinic acid)-mononucleotide by PncB (Johnson et al., 2015). Nicotinate mononucleotide is then converted to nicotinic acid adenine dinucleotide by NadD, whereafter NadE converts nicotinic acid adenine dinucleotide to nicotine adenine dinucleotide (NAD) (Johnson et al., 2015). Another important enzyme glyceraldehyde-3-phosphate dehydrogenase (GAP) is a highly conserved and a multifunctional protein with significant activity in several fundamental cell pathways (Sirover, 2011). Usually, the dehydrogenase reactions of metabolic pathways have been deemed the major sources of NADPH. Nevertheless, the importance of transhydrogenases, glucose dehydrogenases, and non-phosphorylating glyceraldehyde 3- phosphate dehydrogenase (GAPN), is becoming eminent, suggesting that the traditional view is over-simplistic (Sauer U. et al., 2004; Matsubara et al., 2011; Bräsen et al., 2014). As NAD is a vital cofactor used by all living organisms, all bacterial species make use of the pathways to reduce NAD^+^ to NADH (Jurtshuk, 1996). NAD^+^ is also used by bacteria as a substrate for dehydrogenases involved in breaking down aldehydes and alcohols (Nobelmann and Lengeler, 1996; Kotrbova-Kozak et al., 2007; Luong et al., 2015). Furthermore, several cellular processes in bacterial and mammalian cells also use NAD, for instance DNA ligation and repair, redox recycling in the pyruvate dehydrogenase pathway, and synthesis of acetyl-CoA for the tricarboxylic acid cycle (Ishino et al., 1986; Satoh and Lindahl, 1992; Wilkinson et al., 2001; Chalkiadaki and Guarente, 2012; Chiarugi et al., 2012; Patel et al., 2014).

YrxA (NiaR) was found to be a niacin-responsive repressor of NAD *de novo* synthesis in *Bacillus subtilis* and transcriptional regulation of NAD biosynthesis in bacteria having orthologs of *B. subtilis yrxA* was determined using a comparative genomic approach and expression studies (Rodionov et al., 2008a). NiaR family members are generally conserved in the Bacillus/Clostridium group and in the unrelated Fusobacteria and Thermotogales lineages (Rodionov et al., 2008a). The NiaR regulon is not limited to the transcriptional regulation of the *nadABC* but in some species it also covers niacin salvage (the *pncAB* genes) and contains uncharacterized membrane proteins putatively involved in niacin transport (Rodionov et al., 2008a). Moreover, members of the NiaP family (involved in niacin uptake) are not only conserved in bacteria but also in multicellular eukaryotes, including humans, suggesting the putative involvement of NiaP in niacin utilization in these organisms (Rodionov et al., 2008a).

This study explains the transcriptomic response of *S. pneumoniae* D39 to niacin and regulation of *niaX, pnuC*, and *nadC* genes. We established that the transcriptional regulator NiaR acts as a transcriptional repressor for *niaX, pnuC*, and *nadC* genes involved in niacin uptake and utilization. The putative operator site (5′-TACWRGTGTMTWKACASYTRWAW-3′ where R = A/G, K = G/T, S = G/C, Y = T/C, W = A/T and M = A/C) of NiaR in the promoter regions of *niaX, pnuC*, and *nadC* is predicted, and subsequently confirmed by mutating NiaR operator sites in the respective promoters.

## Materials and methods

### Bacterial strains and growth conditions

Bacterial strains and plasmids used in this study are listed in Table 1. *S. pneumonia*e D39 was grown as described previously (Kloosterman et al., 2006; Afzal et al., 2014). For β-galactosidase assays, derivatives of *S. pneumoniae* D39 were grown in chemically defined medium (CDM) (Neves et al., 2002) with or without 10 mM niacin. CDM was prepared without niacin. For selection on antibiotics, media were supplemented with the following concentrations of antibiotics: 150 μg/ml spectinomycin and 2.5 μg/ml tetracycline for *S. pneumoniae*, and 100 μg/ml ampicillin for *E. coli*. All bacterial strains used in this study were stored in 10% (v/v) glycerol at −80°C. For PCR amplification, chromosomal DNA of *S. pneumoniae* D39 (Lanie et al., 2007) was used as a template. Primers used in this study are based on the sequence of the *S. pneumoniae* D39 genome and listed in Table 2.

**Table 1:** **List of strains and plasmids used in this study**.

**Strain/plasmid**	**Description**	**Source**
***S. PNEUMONIAE***		
D39	Serotype 2 strain. *cps 2*	Laboratory of P. Hermans.
MA1300	D39 Δ*niaR*; Spec^R^	This study
MA1301	D39 Δ*bgaA*:: P*niaX-lacZ*; Tet^R^	This study
MA1302	D39 Δ*bgaA*:: P*pnuC-lacZ*; Tet^R^	This study
MA1303	D39 Δ*bgaA*:: P*nadC-lacZ*; Tet^R^	This study
MA1304	MA1300 Δ*bgaA*:: P*niaX-lacZ*; Tet^R^	This study
MA1305	MA1300 Δ*bgaA*:: P*pnuC-lacZ*; Tet^R^	This study
MA1306	MA1300 Δ*bgaA*:: P*nadC-lacZ*; Tet^R^	This study
MA1307	D39 Δ*bgaA*:: P*niaX-M-lacZ*; Tet^R^	This study
MA1308	D39 Δ*bgaA*:: P*nuC-M-lacZ*; Tet^R^	This study
MA1309	D39 Δ*bgaA*:: P*nadC-R1-M-lacZ*; Tet^R^	This study
MA1310	D39 Δ*bgaA*:: P*nadC-R2-M-lacZ*; Tet^R^	This study
MA1311	D39 Δ*bgaA*:: P*fba-lacZ*; Tet^R^	This study
MA1312	D39 Δ*bgaA*:: P*rex-lacZ*; Tet^R^	This study
MA1313	D39 Δ*bgaA*:: P*gapN-lacZ*; Tet^R^	This study
MA1314	D39 Δ*bgaA*:: P*pncB-lacZ*; Tet^R^	This study
MA1315	D39 Δ*bgaA*:: P*gap-lacZ*; Tet^R^	This study
MA1316	D39 Δ*bgaA*:: P*adhE-lacZ*; Tet^R^	This study
MA1317	D39 Δ*bgaA*:: P*adhB2-lacZ*; Tet^R^	This study
***E. COLI***		
EC1000	Km^R^; MC1000 derivative carrying a single copy of the pWV1 *repA* gene in *glgB*	Laboratory collection
**PLASMIDS**		
pPP2	Amp^R^ Tet^R^; promoter-less *lacZ*. For replacement of *bgaA* with promoter *lacZ* fusion. Derivative of pPP1	Halfmann et al., 2007
pMA1301	pPP2 P*niaX-lacZ*	This study
pMA1302	pPP2 P*pnuC-lacZ*	This study
pMA1303	pPP2 P*nadC-lacZ*	This study
pMA1304	pPP2 P*niaX-M-lacZ*	This study
pMA1305	pPP2 P*nuC-M-lacZ*	This study
pMA1306	pPP2 P*nadC-R1-M-lacZ*	This study
pMA1307	pPP2 P*nadC-R1-M-lacZ*	This study
pMA1308	pPP2 P*fba-lacZ*	This study
pMA1309	pPP2 P*rex-lacZ*	This study
pMA1310	pPP2 P*gapN-lacZ*	This study
pMA1311	pPP2 P*pncB-lacZ*	This study
pMA1312	pPP2 P*gap-lacZ*	This study
pMA1313	pPP2 P*adhE-lacZ*	This study
pMA1314	pPP2 P*adhB2-lacZ*	This study

**Table 2:** **List of primers used in this study**.

**Name**	**Nucleotide Sequence (5′ → 3′)**	Restriction site^*^
niaX-F	CATGGAATTCTCAAACCTGAAGGTGGAGAT	*EcoRI*
niaX-R	CATGGGATCCGCATAACAATTGGAATCAAAATCG	*BamHI*
pnuC-F	CATGGAATTCCCATATGATTCTTTCTAATGAGTTG	*EcoRI*
pnuC-R	CATGGGATCCGCAAATAAGTATGCATCATTTCTCC	*BamHI*
nadC-F	CATGGAATTCCCAATGGCTAGAGCAATGGC	*EcoRI*
nadC-R	CATGGGATCCCATCTTCTCGCAAGGCTGC	*BamHI*
niaX-M-R	CATGGGATCCCACAAGAATCTCCTTTTTAACGGCATATGTACTAGTATGG	*BamHI*
pnuC-M-F	CATGGAATTCCATGATTTTCTAAAATTTTACTACAAAGACGGTTGAC	*EcoRI*
nadC-R1-M-F	CATGGAATTCGACTATTATACACAAAAAAAATACAATTACCTTGACCATTGTA	*EcoRI*
nadC-R2-M-F	CATGGAATTCTACACAAAAAAAATACAATTGTCTTGACAATTACATTGACCCTTGTT	*EcoRI*
NiaR-1	GCCATGTTCTTGTCGCCC	-
NiaR-2	GCATAGGCGCGCCCAAGAGTTGGAGCAGGGC	*AscI*
NiaR-3	CGATTGCGGCCGCGCCGAAACACAACAAGACC	*NotI*
NiaR-4	CGCTGGTCTGGTTATGCC	-
fba-F	CATGGAATTCCGTCCAAGACTAGGGAGAG	*EcoRI*
fba-R	CATGGGATCCGCATAACCGTTGTCACGGG	*BamHI*
rex-F	CATGGAATTCCCTCATGGATAGCTTGGTAG	*EcoRI*
rex-R	CATGGGATCCGCTGTAGCTTTTGGAATAGC	*BamHI*
gapN-F	CATGGAATTCGGTTTGGCTGTCCCCAACC	*EcoRI*
gapN-R	CATGGGATCCGTCATGGCTGGAACTGTACC	*BamHI*
pncB-F	CATGGAATTCGCTATGGCGAATGGGCTC	*EcoRI*
pncB-R	CATGGGATCCCTGGTACAAGTCCGTGTGC	*BamHI*
gap-F	CATGGAATTCCGTTACGCTATGAATAATAAGGG	*EcoRI*
gap-R	CATGGGATCCCGACCGATACGTCCGAAACC	*BamHI*
adhE-F	CATGGAATTCGCGCTTACCTGTAAATCCC	*EcoRI*
adhE-R	CATGGGATCCGAACCAACTCATCTACGTGC	*BamHI*
adhB2-F	CATGGAATTCGCAACCTACCTAGATGGCG	*EcoRI*
adhB2-R	CATGGGATCCGCACAATAGCGTCTGTTGGC	*BamHI*
NiaR-Conf-1	GGAGATTCTTGTGAATACACGG	-
NiaR-Conf-2	GATAATATCTCTGGTAGTAAGTCTG	-
Spec-R	GCTAAGCGGCCGCACTAAACGAAATAAACGC	*NotI*
Spec-F	GCTATGGCGCGCCCTAATCAAAATAGTGAGGAGG	*AscI*

* *Restriction sites are underlined*.

### Construction of a *niaR* mutant

A *niaR* mutant (MA1300) was constructed in *S. pneumoniae* D39 by allelic replacement with a spectinomycin-resistance cassette. Primer pairs niaR-1/niaR-2 and niaR-3/niaR-4 were used to generate PCR fragments of the left and right flanking regions of *niaR* using Phusion® High-Fidelity DNA polymerase. PCR products of left and right flanking regions of *niaR* contain *AscI* and *NotI* sites, respectively. The spectinomycin-resistance marker, which was amplified by primers SpecR/SpecF from pORI38, also contains *AscI* and *NotI* sites on its ends. Then, by restriction and ligation, the left and right flanking regions of *niaR* were fused to the spectinomycin-resistance gene. The resulting ligation products were transformed to *S. pneumoniae* D39 wild-type and selection of the mutant was done on the appropriate concentration of spectinomycin. Deletion of *niaR* was further verified by PCR using primer pair NiaR-Conf-1/NiaR-Conf-2 and DNA sequencing.

### Construction of promoter *lacZ*-fusions and their use in β-galactosidase assays

Chromosomal transcriptional *lacZ*-fusions to *niaX, pnuC*, and *nadC* promoters were constructed in pPP2 (Halfmann et al., 2007) with primer pairs mentioned in Table 2, resulting in pMA1301-03, respectively. These constructs were further introduced into D39 wild-type and D39 Δ*niaR* (MA1300) resulting in strains MA1301-03 and MA1304-06, respectively. The following *lacZ*-fusions of P*niaX*, P*pnuC*, and P*nadC* with mutations in the NiaR site were made in pPP2 (Halfmann et al., 2007) using the primer pairs mentioned in Table 2: P*niaX-M* (mutation in the *niaR* site), P*pnuC-M* (mutation in the *niaR* site), P*nadC-R1* (mutation in the *niaR* site 1), and P*nadC-R2* (mutation in the *niaR* site 2), resulting in plasmids pMA1304-07, respectively. These constructs were introduced into the *S. pneumoniae* D39 wild-type strain, resulting in strains MA1307-1310, respectively. Similarly, chromosomal transcriptional *lacZ*-fusions to *fba, rex, gapN, pncB, gap, adhE*, and *adhB2* promoters were constructed in pPP2 (Halfmann et al., 2007) with primer pairs mentioned in Table 2, resulting in pMA1308-14, respectively. These constructs were further introduced into D39 wild-type resulting in strains MA1311-17, respectively. All plasmid constructs were further checked for the presence of the right insert by PCR and DNA sequencing.

β-galactosidase assays were performed as described before (Israelsen et al., 1995; Halfmann et al., 2007) using cells that were harvested in the mid-exponential growth phase, and grown in CDM (Neves et al., 2002) with or without niacin as mentioned in the results section.

### Microarray analysis

Microarray analysis was performed as described before (Afzal et al., 2015; Shafeeq et al., 2015). For DNA microarray analysis of *S. pneumoniae* in the presence of niacin, the transcriptome of *S. pneumoniae* D39 wild-type grown in replicates in CDM with 10 mM niacin was compared to that grown in CDM with 0 mM niacin and harvested at their respective mid-exponential growth phases.

For DNA microarray analysis of D39 Δ*niaR*, the transcriptome of *S. pneumoniae* D39 Δ*niaR* was compared to *S. pneumoniae* D39 wild-type grown in replicates in complete CDM and harvested at respective mid-exponential growth phases. Complete CDM contains 8 μM of niacin. The procedures for DNA microarray analysis were performed as described previously (Afzal et al., 2015; Shafeeq et al., 2015). For the identification of differentially expressed genes, a Bayesian *p* < 0.001 and a fold-change cut-off > 1.5 was applied. Microarray data have been submitted to GEO (Gene Expression Omnibus) under accession numbers GSE94511 and GSE94513.

## Results

### Niacin-dependent gene regulation in *S. pneumoniae* D39

Microarray comparison of *S. pneumoniae* D39 grown in CDM with 0 mM to same strain grown in CDM with 10 mM niacin was performed to explore the impact of niacin on the transcriptome of *S. pneumoniae* D39 wild-type. CDM was prepared without niacin. A number of genes/operons were differentially expressed under our tested conditions (Table 3). A particular gene cluster (*spd-0093-0095*) was significantly upregulated in the absence of niacin. This gene cluster codes for three hypothetical proteins, which are putative membrane proteins. Another gene cluster (*spd-1798-1802*) was significantly upregulated in the absence of niacin. This gene cluster consists of a DNA-binding response regulator (encoded by *spd-1798*), a sensor histidine kinase (encoded by *spd-1799*), two hypothetical proteins (encoded by *spd-1800* and *spd-1802*) and an ABC transporter (encoded by *spd-1801*). Some genes that appear to be a part of a gene cluster were also downregulated under our tested conditions (*spd-0113-15* and *spd-0122-24*). All of these genes code for hypothetical proteins and the role of these genes warrants further investigation.

**Table 3:** **Summary of the transcriptome comparison of *S. pneumoniae* D39 wild-type grown in CDM with 0 mM niacin to grown in CDM with 10 mM niacin**.

**D39 tag^a^**	**Function^b^**	**Ratio^c^**
**UPREGULATED GENES**		
*spd_0093*	Hypothetical protein	3.1
*spd_0094*	Hypothetical protein	2.8
*spd_0095*	Hypothetical protein	2.4
*spd_0474*	Hypothetical protein	4.6
*spd_0475*	CAAX amino terminal protease family protein	3.5
*spd_0526*	Fructose-1,6-bisphosphate aldolase, class II, Fba	1.5
*spd_0976*	Redox-sensitive transcriptional regulator Rex	1.5
*spd_1004*	Glyceraldehyde-3-phosphate dehydrogenase, NADP-dependent, GapN	3.5
*spd_1091*	Substrate-specific component predicted niacin ECF transporter, NiaX	1.8
*spd_1250*	NAD^+^ synthetase, NadE	1.5
*spd_1251*	Nicotinate phosphoribosyltransferase, putative, PncB	1.9
*spd_1640*	Ribosyl nicotinamide transporter, PnuC-like, PnuC	4.2
*spd_1798*	DNA-binding response regulator	2.1
*spd_1799*	Sensor histidine kinase, putative	2.0
*spd_1800*	Hypothetical protein	2.4
*spd_1801*	ABC transporter, ATP-binding protein	2.0
*spd_1802*	Hypothetical protein	2.2
*spd_1823*	Glyceraldehyde-3-phosphate dehydrogenase, type I, Gap	1.7
*spd_1824*	Hypothetical protein	2.2
*spd_1826*	Nicotinate-nucleotide pyrophosphorylase, NadC	4.4
*spd_1827*	Hypothetical protein	3.1
*spd_1833*	PTS system, IIA component	1.7
*spd_1834*	Alcohol dehydrogenase, iron-containing, AdhE	5.8
*spd_1865*	Alcohol dehydrogenase, zinc-containing, AdhB2	1.7
*spd_1874*	LysM domain protein	3.7
**DOWNREGULATED GENES**		
*spd_0113*	Hypothetical protein	−2.9
*spd_0114*	Hypothetical protein	−3.1
*spd_0115*	Hypothetical protein	−2.7
*spd_0122*	Hypothetical protein	−2.2
*spd_0123*	Hypothetical protein	−2.4
*spd_0124*	Hypothetical protein	−2.0

a *Gene numbers refer to D39 locus tags*.

b *D39 annotation/TIGR4 annotation (Lanie et al., 2007)*.

c *Ratio represents the fold increase/decrease in the expression of genes in CDM with 0 mM Niacin to CDM with 10 mM Niacin. Errors in the ratios never exceeded 10% of the given values*.

Putative niacin biosynthesis pathway genes were significantly upregulated in the absence of niacin (*fba, rex, gapN, niaX, pncB*-*nadE, pnuC, gap, spd-1824, nadC, adhE*, and *adhB2*). *fba* codes for a fructose-bisphosphate aldolase, whereas *rex* encodes a redox-sensitive transcriptional regulator. Similarly, *gapN* encodes a glyceraldehyde-3-phosphate dehydrogenase that is involved in generation of NADPH from NADH. *pncB* encodes a nicotinate phosphoribosyltransferase that converts nicotinate into nicotinate D-ribonucleotide and *vice versa*, whereas *nadE* encodes a NAD^+^ synthetase that converts deamino-NAD^+^ to NAD^+^ and *adhE* codes for an alcohol dehydrogenase. *gap* encodes another glyceraldehyde-3-phosphate dehydrogenase and *adhE* codes for an iron-containing alcohol dehydrogenase, whereas *adhB2* encodes a zinc-containing alcohol dehydrogenase. NiaX (encoded by *niaX*) is a substrate-specific component predicted niacin ECF transporter, whereas PnuC (encoded by *pnuC*) is a ribosyl nicotinamide transporter. NadC (encoded by *nadC*) is a nicotinate-nucleotide pyrophosphorylase and has been proposed to convert quinolinate formed from alanine, aspartate, and glutamate, and tryptophan metabolism into nicotinate D-ribonucleotide (Kanehisa et al., 2014).

### Niacin-dependent expression of *fba, rex, gapN, niaX, pncB, pnuC, gap, spd-1824, nadC, adhE*, and *adhB2*

**Figure 1:**
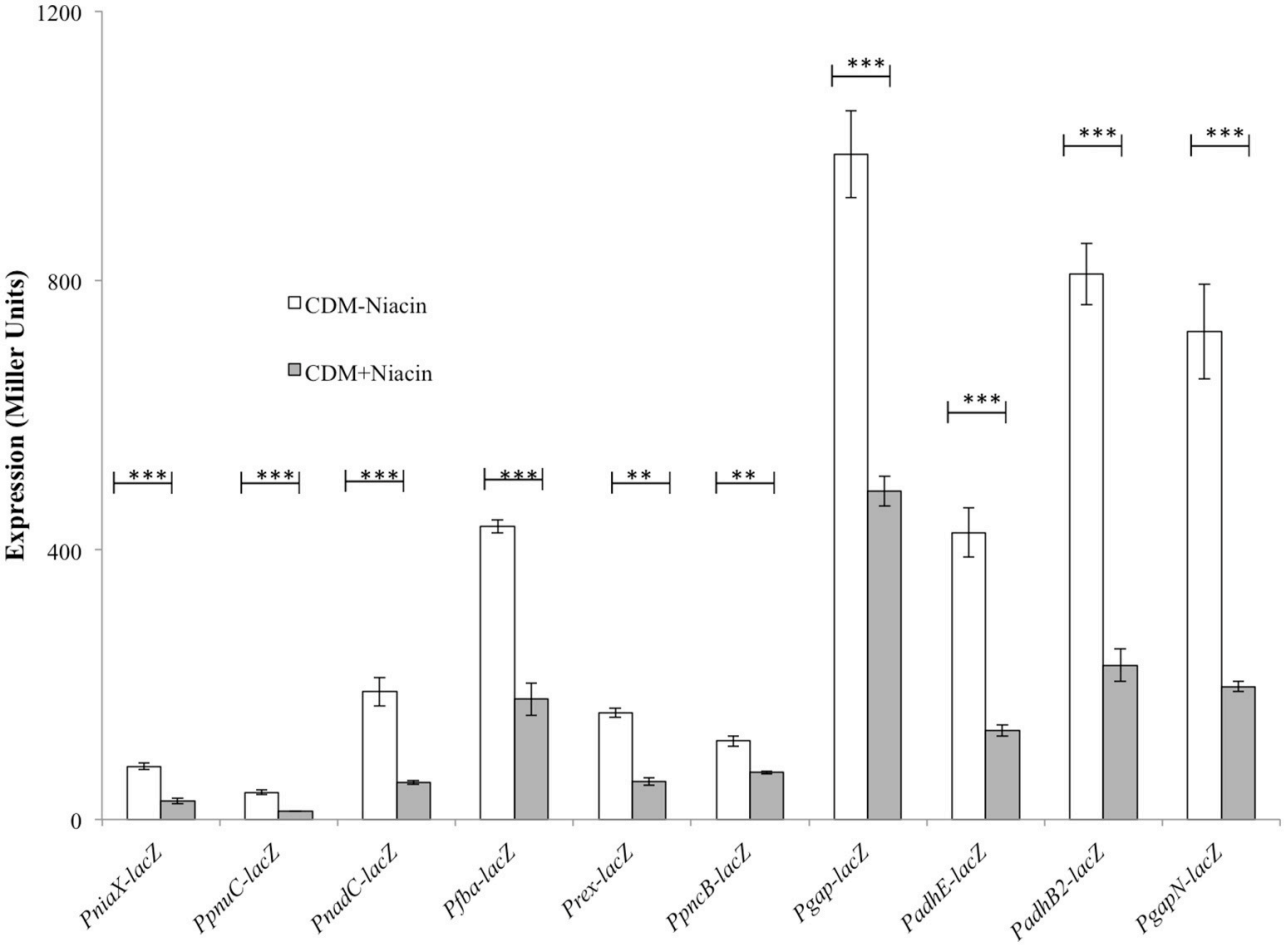
**Expression levels (in Miller units) of P*niaX-lacZ*, P*pnuC-lacZ*, P*nadC-lacZ*, P*fba-lacZ*, P*rex-lacZ*, P*pncB-lacZ*, P*gap-lacZ*, P*adhE-lacZ*, P*adhB2-lacZ*, and P*gapN*-*lacZ* in CDM with 0 and 10 mM niacin in *S. pneumoniae* D39 wild-type**. Standard deviations of three independent experiments are indicated in bars. Statistical significance of the differences in the expression levels was determined by one-way ANOVA (NS, not significant, ^**^P < 0.001, and ^***^P < 0.0001).

Our niacin-dependent microarray data mentioned above indicated the role of niacin in the regulation of *fba, rex, gapN, niaX, pncB, pnuC, gap, spd-1824, nadC, adhE*, and *adhB2*. To confirm our microarray results and further study the effect of niacin on the expression of *fba, rex, gapN, niaX, pncB, pnuC, gap, spd-1824, nadC, adhE*, and *adhB2*, we performed β-galactosidase assays with promoter *lacZ*-fusions of these genes constructed in *S. pneumoniae* D39 wild-type. Our β-galactosidase data demonstrated that the expression of P*fba-lacZ*, P*rex*-*lacZ*, P*gapN*-*lacZ*, P*niaX*-*lacZ*, P*pncB*-*lacZ*, P*pnuC*-*lacZ*, P*gap*-*lacZ*, P*nadC*-*lacZ*, P*adhE*-*lacZ*, and P*adhB2*-*lacZ* increased significantly in the absence of niacin in the medium (Figure 1). These data further confirm our microarray data described above and suggest the role of niacin in the regulation of these genes.

### Microarray analysis of D39 Δ*niaR*

Niacin genes are mostly regulated by a transcriptional regulator NiaR in different bacteria (Novichkov et al., 2010). In Firmicutes and Thermotogales, transcriptional regulator NiaR regulates the NAD biosynthesis and salvage of niacin (Rodionov et al., 2008a). NiaR was first studied in *B. subtilis* as a niacin-responsive transcriptional repressor that binds to its DNA targets in the presence of niacin (Rossolillo et al., 2005). NiaR belongs to a unique protein family, which possesses an N-terminal HTH (Helix-Turn-Helix) DNA binding domain (PF08279) and a C-terminal effector binding domain, called the 3H domain (PF02829). *S. pneumoniae* also possesses a NiaR transcriptional regulator, which might be involved in the regulation of the niacin-regulated genes described above. Therefore, we decided to further study the role of NiaR in the regulation of these genes.

A deletion mutant of the *niaR* gene was constructed and microarray comparison of *S. pneumoniae* D39 Δ*niaR* to D39 wild-type grown in complete CDM was performed to investigate the role of NiaR in *S. pneumoniae* D39. Complete CDM contains 8 μM of niacin. Table 4 summarizes the transcriptome changes induced by the deletion of *niaR* in *S. pneumoniae* D39. Expression of *niaR* was downregulated about 3-fold confirming the *niaR* deletion in D39 Δ*niaR*. Expression of *nadC, niaX*, and *pnuC* was upregulated significantly in D39 Δ*niaR*, suggesting the role of NiaR as a transcriptional repressor of *niaX, nadC*, and *pnuC* in *S. pneumoniae* D39. Expression of *spd-1824* and *spd-1827* (coding for hypothetical proteins) was also upregulated. *Spd*-*1827* is localized adjacent to *nadC* (*spd-1826*), but transcribed in opposite direction.

**Table 4:** **Summary of transcriptome comparison of *S. pneumoniae* D39 Δ*niaR* compared to the D39 wild-type grown in complete CDM**.

**D39 tag^a^**	**Function^b^**	**Ratio^c^**
*spd_1091*	Substrate-specific component predicted niacin ECF transporter, NiaX	2.1
*spd_1093*	Transcriptional regulator, biotin repressor family protein, NiaR	−2.7
*spd_1640*	Ribosyl nicotinamide transporter, PnuC-like, PnuC	1.5
*spd_1824*	Hypothetical protein	3.5
*spd_1826*	Nicotinate-nucleotide pyrophosphorylase, NadC	7.2
*spd_1827*	Hypothetical protein	3.1

*Complete CDM contains 8 μM of niacin*.

a *Gene numbers refer to D39 locus tags*.

b *D39 annotation/TIGR4 annotation (Lanie et al., 2007)*.

c *Ratio represents the fold increase/decrease in the expression of genes in D39 ΔniaR compared to the D39 wild-type in complete CDM. Errors in the ratios never exceeded 10% of the given values*.

### Role of NiaR as a transcriptional repressor of *niaX, nadC*, and *pnuC*

**Figure 2:**
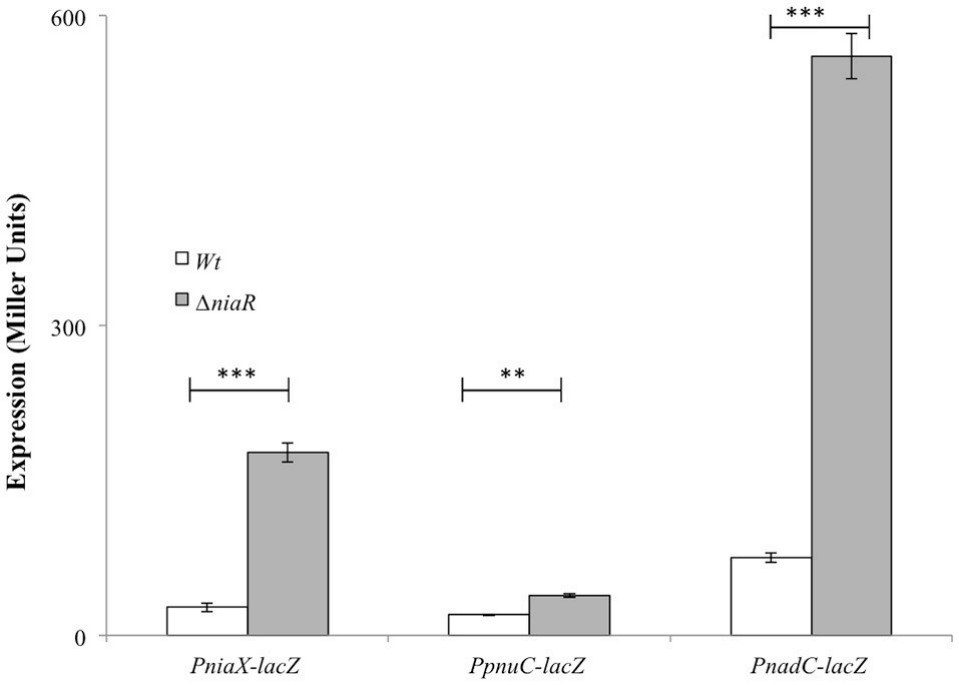
**Expression levels (in Miller units) of P*niaX-lacZ*, P*pnuC-lacZ*, and P*nadC*-*lacZ* in complete CDM in *S. pneumoniae* D39 wild-type and D39 Δ*niaR***. Standard deviations of three independent experiments are indicated in bars. Statistical significance of the differences in the expression levels was determined by one-way ANOVA (NS, not significant, ^**^P < 0.001, and ^***^P < 0.0001).

To further investigate the role of NiaR in the regulation of *niaX, nadC*, and *pnuC*, we transformed the *lacZ*-fusions of the promoter regions of *niaX, nadC*, and *pnuC* into D39 Δ*niaR* and performed β-galactosidase assays in complete CDM (Figure 2). The results of the β-galactosidase assays showed that the activity of all these promoters increased significantly in D39 Δ*niaR* compared to the D39 wild-type, confirming the role of NiaR as a transcriptional repressor of *niaX, nadC*, and *pnuC*.

### Prediction and confirmation of the NiaR site in P*niaX*, P*nadC*, and P*pnuC*

**Figure 3:**
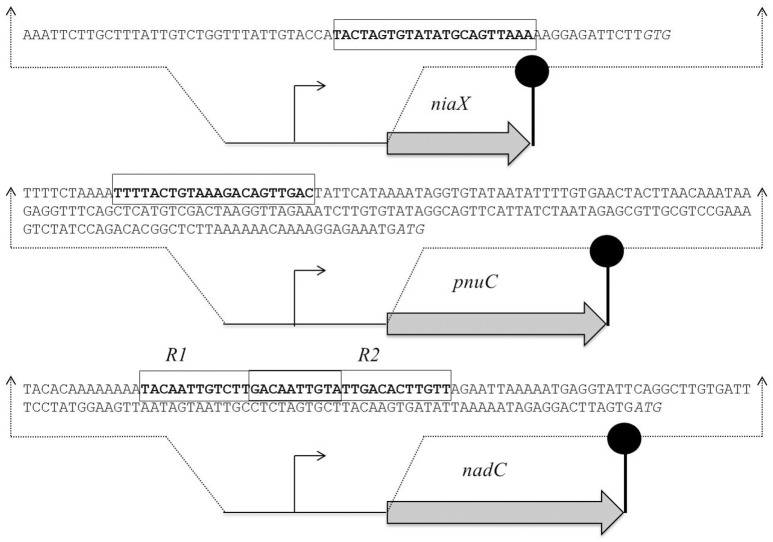
**Organization of the NiaR-regulated genes in *S. pneumoniae* D39**. Putative NiaR operator sequences are rectangle and translational initiation sites are italicized, whereas the lollipop structures represent the putative transcriptional terminators. See text for further details.

**Figure 4:**
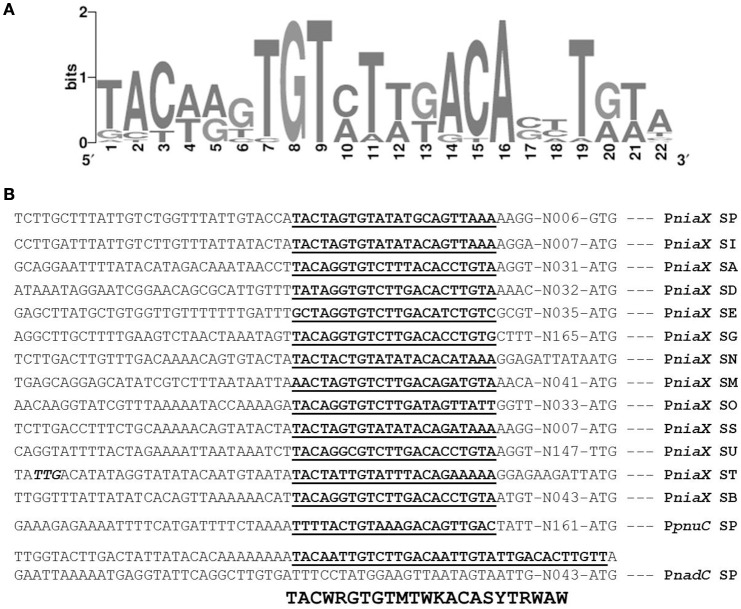
**Identification of the NiaR operator site. (A)** Weight matrix of the identified NiaR operator site in the promoter regions of *niaX, nadC*, and *pnuC*. **(B)** Position of the NiaR operator site in the promoter region of *niaX, nadC*, and *pnuC* in different streptococci. Putative NiaR operator sites are bold and underlined. SP, *S. pneumoniae*; SI, *Streptococcus mitis*; SA, *Streptococcus agalactiae*; SD, *Streptococcus dysgalactiae*; SE, Streptococcus equi; SG, *Streptococcus gallolyticus*; SN, *Streptococcus gordonii*; SM, *Streptococcus mutans*; SO, *Streptococcus pyogenes*; SS, *Streptococcus sanguinis*; SU, *Streptococcus suis*; ST, *Streptococcus thermophiles*; and SB, *Streptococcus uberis*.

The promoter regions of all of the upregulated genes, including *spd_1824* and *spd_1827*, were analyzed by Genome2D software (Baerends et al., 2004) and a MEME motif sampler search (Bailey and Elkan, 1994). A 22-bp palindromic-like sequence was found in the promoter regions of *niaX, nadC*, and *pnuC* (Figure 3). This DNA sequence might serve as the NiaR operator site in *S. pneumoniae*. P*niaX* from different streptococci was also analyzed for the presence of NaiR site. The NiaR site present in the promoter region of *niaX* of different streptococci is shown in Figure 4. Weight matrix based on these putative NiaR sites (5′- TACWRGTGTMTWKACASYTRWAW -3′) was constructed (Figure 4).

**Figure 5:**
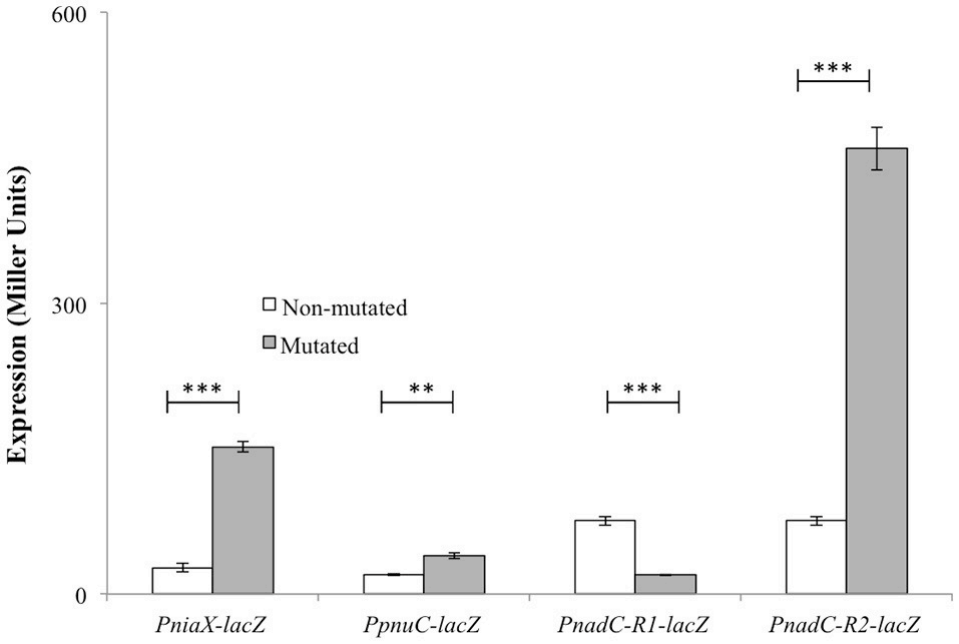
**Expression levels (in Miller units) of P*niaX-lacZ*, P*pnuC-lacZ*, and P*nadC-lacZ* with mutated and non-mutated NiaR operator sites in *S. pneumoniae* D39 wild-type grown in complete CDM**. Standard deviations of three independent experiments are indicated in bars. Statistical significance of the differences in the expression levels was determined by one-way ANOVA (NS, not significant, ^**^P < 0.001, and ^***^P < 0.0001).

The predicted NiaR operator site present in the promoter regions of *niaX, nadC*, and *pnuC* was further verified by promoter mutational experiment. For this purpose, we made transcriptional *lacZ*-fusions of P*niaX*, P*pnuC*, and P*nadC*, where conserved bases in the putative NiaR sites were mutated in P*niaX* (5′- TACTAGT**GT**ATATGC**A**GTTAAA-3′ to 5′- TACTAGT**AC**ATATGC**C**GTTAAA -3′), P*pnuC* (5′- TTTTACT**GT**AAAGAC**A**GTTGAC -3′ to 5′- TTTTACT**AC**AAAGAC**G**GTTGAC -3′), P*nadC-R1* (5′- TACAATT**GT**CTTGAC**A**ATTGTA -3′ to 5′- TACAATT**AC**CTTGAC**C**ATTGTA -3′), and P*nadC-R2* (5′- GACAATT**GT**ATTGAC**A**CTTGTT -3′ to 5′- GACAATT**AC**ATTGAC**C**CTTGTT -3′). β-galactosidase assays were performed on cells grown in complete CDM. Complete CDM contains 8 μM of niacin. The expression of P*niaX* and P*pnuC* with mutated conserved bases of NiaR operator sites increased significantly in *S. pneumoniae* D39 wild-type, confirming that the predicted NiaR sites present in the promoter regions of *niaX* and *pnuC* are active and intact in *S. pneumoniae* (Figure 5). Two putative operator sites for NiaR are present in P*nadC* (R1 and R2). We mutated both sites individually and performed β-galactosidase assays. We could only observe derepression (caused by NiaR) in the activity of P*nadC* when NiaR operator site 2 (R2) was mutated and did not observe any change in the activity of P*nadC* due to mutation in NiaR operator site 1 (R1) (Figure 5). These data suggest that operator site 2 (R2) is the functional operator site in P*nadC*.

## Discussion

NAD is an essential cofactor used by all living organisms. NAD synthesis is a tightly regulated intracellular process in bacteria (Huang et al., 2009). Bacteria acquire NAD in two main ways: through *de novo* synthesis and through the salvage pathway. Some bacteria do not have the ability to *de novo* synthesize NAD and must make use of the salvage pathway to import niacin or nicotinamide riboside through the substrate importers NiaX and PnuC, respectively. The *de novo* pathway synthesizes NAD from aspartic acid, whereas the salvage pathway brings intermediates many steps downstream into the NAD *de novo* synthesis pathway (Rodionov et al., 2008b). NiaX and PnuC are the two major importers in the NAD salvage pathway, where NiaX is responsible for niacin uptake, and PnuC transports nicotinamide riboside (Herbert et al., 2003; Sauer E. et al., 2004; Rodionov et al., 2008a, 2009). Our current study demonstrates the transcriptomic response of *S. pneumoniae* to niacin and reveals that a number of genes including *pnuC, pncB*, and *nadC* are differentially expressed under the tested conditions. We further demonstrate that a transcriptional regulator NiaR acts as a transcriptional repressor of *niaX, pnuC*, and *nadC* in the presence of niacin.

An extracellular protein capable of modifying nicotinamide mononucleotide to an importable form appears to help NiaX and PnuC for importing nicotinamide mononucleotide or there may be another import system in *S. pneumoniae* (Johnson et al., 2015). There is significant variability between PnuC homologs (Jaehme et al., 2014), and the PnuC homologs from *Haemophilus influenzae* and *Salmonella typhimurium* do not import nicotinamide mononucleotide, but can transform it to an importable form for PnuC (nicotinamide riboside) through NadN or AphA, respectively (Kemmer et al., 2001; Grose et al., 2005). The PnuC proteins from *H. influenzae, S. typhimurium*, and *S. pneumoniae* all possess the motif for nicotinamide mononucleotide binding. Nevertheless, PnuC homologs from many other organisms lack the consensus binding residues (Kemmer et al., 2001; Sauer E. et al., 2004; Grose et al., 2005). These observations indicate that different groups of NAD salvage substrate importers (annotated as PnuC) import nicotinamide riboside and/or nicotinamide mononucleotide, and that NiaX imports niacin and/or nicotinamide mononucleotide as preferred substrates. Moreover, the amino acids in *Salmonella* PnuC curtailing import of nicotinamide mononucleotide are not conserved in the pneumococci, suggesting that the pneumococcal PnuC may permit this substrate along with nicotinamide riboside. Although, both PnuC and NiaX in *S. pneumoniae* may have acquired the ability to import nicotinamide mononucleotide, an extra importer (that is yet to be characterized) may also be present (Johnson et al., 2015). The role of PnuC in pneumococcal pathogenesis has been studied and PnuC could be a potential viable small molecule therapeutic target to halt disease progression in the host (Johnson et al., 2015). The proposed NAD pathway in *S. pneumoniae* states that niacin and nicotinamide enter the cells through NiaX, and PnuC transports nicotinamide riboside to the inside of the cell, whereas the transporter for nicotinamide mononucleotide is unknown (Johnson et al., 2015). *spd-1411* encodes a nicotinamidase (PncA) that converts nicotinamide into niacin, which is further converted into nicotinate mononucleotide by a nicotinic acid phosphoribosyltransferase (PncB) (Johnson et al., 2015). The nicotinate mononucleotide is then converted into NAD by NadD and NadE. Moreover, NadD (nicotinate/nicotinamide nucleotide adenylyltransferase) converts nicotinamide riboside and nicotinamide mononucleotide into NAD (Johnson et al., 2015). Nicotinamide riboside augmentation has been attributed to several advantageous functions in the host, including shielding against mitochondrial myopathy (Khan et al., 2014), hearing loss (Brown et al., 2014) and obesity (Cantó et al., 2012). These functions may not be due to increasing NAD synthesis (Frederick et al., 2015), but may be due to overall bioavailability. While nicotinamide riboside is required for pathogen and host, luckily pneumococcal PnuC and its homologous in other bacteria do not have sequence homology to any proteins in the animal kingdom. Hence, PnuC could be a potential therapeutic target in bacterial species shielding this pathway without mammalian significance as has been effectively shown with *H. influenzae* (Sauer E. et al., 2004).

NiaR orthologs have been found in 30 out of 45 species from the *Bacillus*/*Clostridium* group (Firmicutes), in addition to the diverged groups of the Fusobacteria and Thermotogales and for the *Bacillus*/*Clostrida* group another DNA binding site was proposed (Rodionov et al., 2008a). There are two different types of DNA-binding sites of NiaR i.e., type I operator found in Firmicutes and Fusobacteria, and type II in the Thermotogales. The niacin-responsive transcription factor NiaR (known as YrxA in *B. subtilis*) was first recognized as a nicotinic acid-responsive repressor of the *de novo* NAD biosynthesis operon (*nadABC*) in *B. subtilis* (Rossolillo et al., 2005). NiaR regulation of the niacin salvage genes *pncB* (in *Lactobacillus plantarum*), *pncA* (in *Streptococcus pyogenes, Streptococcus equi*, and *Clostridium tetani*), and/or the RNam salvage transporter *pnuC* (in *S. pneumoniae* and *Streptococcus mutans*) (Rodionov et al., 2008a) is less common. Moreover, the NiaR regulon contains membrane proteins that putatively have a role in niacin uptake. The most abundant NiaP family is found in ten NiaR-containing species (Bacilli, Lactobacilli and Thermotogales) in addition to several species that do not have the NiaR regulator (Rodionov et al., 2008a). Among Streptococci and Clostridia, NiaX is found in twelve genomes, and NiaY is found in five genomes (Bacilli and Clostridia). Several lines of genomic evidence support the putative involvement of these gene families in niacin uptake including the predicted co-regulation with NAD biosynthesis and niacin salvage genes, and co-occurrence with the niacin salvage genes *pncB*-*pncA* (Rodionov et al., 2008a). Our study demonstrates that *niaX, pnuC*, and *nadC* are the genes that have a putative NiaR operator site in their promoter regions and are repressed by NiaR in the presence of niacin. We have further confirmed the NiaR operator sites in the promoter regions of *niaX, pnuC*, and *nadC* by mutagenesis studies. There are some other genes that are differentially expressed under our tested conditions (*fba, rex, gapN, pncB*-*nadE, gap, spd-1824, spd-1827, adhE*, and *adhB2*). The change in the expression of these genes suggests that these genes may have a role in the transport and biosynthesis of niacin or they may be upregulated due to some indirect effect of niacin genes. These genes do not have a putative NiaR operator site in their promoter regions suggesting the role of another transcriptional regulator in the regulation of *fba, rex, gapN, pncB*-*nadE, gap, spd-1824, spd-1827, adhE*, and *adhB2*. Therefore, we propose that the study of the regulatory mode of the above-mentioned genes would shed light on this possibility.

## Author contributions

Substantial contributions to the conception or design of the work; or the acquisition, analysis, or interpretation of data for the work: MA, SS, and OK. Drafting the work or revising it critically for important intellectual content: MA, SS, and OK. Final approval of the version to be published: MA, SS, and OK. Agreement to be accountable for all aspects of the work in ensuring that questions related to the accuracy or integrity of any part of the work are appropriately investigated and resolved: MA, SS, and OK.

### Conflict of interest statement

The authors declare that the research was conducted in the absence of any commercial or financial relationships that could be construed as a potential conflict of interest.
